# DOES NEOADJUVANT CHEMORADIOTHERAPY FOR ESOPHAGEAL AND GASTROESOPHAGEAL JUNCTION CANCER PATIENTS AFFECT POSTOPERATIVE OUTCOMES? A STUDY USING THE BECKER TUMOR REGRESSION GRADE SYSTEM AND LYMPH NODE REGRESSION

**DOI:** 10.1590/0102-672020230002e1724

**Published:** 2023-05-05

**Authors:** Maria Inês Vaz do Rosário, José Pedro Barbosa, Irene Gullo, José Barbosa

**Affiliations:** 1 Universidade do Porto, Faculty of Medicine – Porto, Portugal; 2 Universidade do Porto, Faculty of Medicine, Information and Decision in Health, Department of Community Medicine – Porto, Portugal; 3 Centro Hospitalar Universitário de São João, Department of Pathology – Porto, Portugal; 4 Centro Hospitalar Universitário de São João, Department of General Surgery – Porto, Portugal.

**Keywords:** Esophageal neoplasms, Drug therapy, Radiotherapy, Limph node, Postoperative complications, Survival, Neoplasias esofágicas, Tratamento farmacológico, Radioterapia, Complicações pós-operatórias, sobrevida

## Abstract

**BACKGROUND:**

The effect of neoadjuvant chemoradiotherapy (nCRT) in patients with locally advanced esophageal cancer can be determined by assessing the Becker tumor regression grade in the primary tumor, as well as in lymph nodes.

**AIMS:**

The aim of this study was to investigate the anatomopathological changes caused by neoadjuvant chemoradiotherapy and their impact on clinical parameters. Specifically, we analyzed the Becker tumor regression grade, lymph node status, and regression changes and evaluated their association with the Clavien-Dindo classification of surgical complications and overall patient survival.

**METHODS:**

This is a retrospective and observational study including 139 patients diagnosed with adenocarcinoma or squamous cell carcinoma of the esophagus and treated with either neoadjuvant chemoradiotherapy followed by surgery or surgery alone. For the 94 patients who underwent neoadjuvant chemoradiotherapy, we evaluated tumor regression by Becker tumor regression grade in primary tumors. We also analyzed lymph node status and regression changes on lymph nodes with or without metastases. Overall survival analysis was performed using Kaplan-Meier curves.

**RESULTS:**

Becker tumor regression grade is associated with lower lymphatic permeation (p<0.01) and vascular invasion (p<0.001), but not with lymph node regression rate (p=0.10). Clavien-Dindo classification was associated neither with lymph node regression rate (odds ratio=0.784, p=0.795) nor with tumor regression grade (p=0.68). Patients who presented with lymphatic permeation and vascular invasion had statistically significantly lower median survival (17 vs. 30 months, p=0.006 for lymphatic permeation, and 14 vs. 29 months, p=0.024 for vascular invasion).

**CONCLUSION:**

In our series, we were unable to demonstrate an association between Becker tumor regression grade and lymph node regression rate with any postoperative complications. Patients with lower lymphatic permeation and vascular invasion have higher overall survival, correlating with a better response in the Becker tumor regression grade system.

## RESUMO

**RACIONAL:**

O efeito da quimioradioterapia neoadjuvante em pacientes com câncer de esôfago localmente avançado pode ser determinado pela avaliação do grau de regressão tumoral de Becker no tumor primário, bem como nos linfonodos.

**OBJETIVOS:**

Investigar as alterações anatomopatológicas causadas pela quimioradioterapia neoadjuvante e seu impacto nos parâmetros clínicos. Especificamente, analisamos o grau de regressão tumoral de Becker, o status linfonodal e as alterações de regressão e avaliamos sua associação com a Classificação Clavien-Dindo de complicações cirúrgicas e a sobrevida geral dos pacientes.

**MÉTODOS:**

Estudo retrospectivo e observacional incluindo 139 pacientes diagnosticados com carcinoma espinocelular de esôfago ou adenocarcinoma da junção esofagogástrica, tratados com quimioradioterapia neoadjuvante seguido de cirurgia ou cirurgia isolada. Para os 94 pacientes submetidos a quimioradioterapia neoadjuvante, avaliamos a grau de regressão tumoral de Becker em tumores primários. Também analisamos o status linfonodal e as alterações de regressão em linfonodos com ou sem metástases. A análise de sobrevida global foi realizada usando curvas de Kaplan-Meier.

**RESULTADOS:**

O grau de regressão tumoral de Becker está associado a menor permeação linfática (p<0,01) e invasão vascular (p<0,001), mas não à taxa de regressão linfonodal (p=0,10). A classificação de Clavien-Dindo não foi associada à taxa de regressão linfonodal (OR=0,784; p=0,795) nem ao grau de grau de regressão tumoral (p=0,68). Os pacientes que apresentavam permeação linfática e invasão vascular tiveram sobrevida mediana menor estatisticamente significativa (17 vs 30 meses; p=0,006 para a permeação linfátiva e 14 vs 29 meses; p=0,024, para a invasão vascular, respectivamente).

**CONCLUSÕES:**

Em nossa série não conseguimos demonstrar associação entre grau de regressão tumoral de Becker e taxa de regressão linfonodal com quaisquer complicações pós-operatórias. Pacientes com menor permeação linfática e invasão vascular apresentam maior sobrevida global, correlacionando-se com uma melhor resposta no sistema Becker.

## INTRODUCTION

Esophageal carcinoma is the sixth leading cause of cancer-related mortality and the seventh most common cancer worldwide. It has an age-standardized rate (ASR) for incidence of 9.3 in males and 3.6 in females and an ASR for mortality of 8.3 in males and 3.2 in females, which represents a two- to threefold difference between sexes in both parameters^21^.

The most common subtype is squamous cell carcinoma (SCC), which represents 90–95% of all cases, and the vast majority occurs in Asia, with China alone representing 54% of the global burden^21^. However, there has been a substantial change in incidence patterns, with decreasing incidence trends in most Asian populations and increasing rates of ADC in historically low-risk populations such as the United Kingdom, the United States, and the Netherlands. This change can be explained by the shift in the prevalence of key risk factors, as decreasing levels of smoking cause a decrease in SCC, and increased prevalence of obesity, gastroesophageal reflux disease, and Barrett's esophagus and decreased chronic infection with *Helicobacter pylori* result in the rising of adenocarcinoma (ADC) incidence^3^.

When it comes to prognosis, it is one of the most aggressive of the gastrointestinal malignancies with an overall 5-year survival rate between 15 and 20% worldwide^16,9^. The early detection rate is low, mainly due to the lack of early clinical symptoms. Most patients are diagnosed with locally advanced disease, which decreases the cure rates and raises the treatment associated morbidity^1^.

Since the CROSS trial (ChemoRadiotherapy for Oesophageal cancer followed by Surgery Study), and according to the ESMO Clinical Practice Guidelines, neoadjuvant chemoradiotherapy (nCRT) has been established as the standard treatment prior to surgery, in patients with SCC or ADC of esophagus or gastroesophageal junction (GEJ) (types I and II – Siewert classification), with locally advanced disease (cT3-T4 or cN1-3 M0)^13,19,23^. Several studies show that patients treated with nCRT show substantial downstaging, reflecting in higher rates of complete surgical resection, fewer positive lymph nodes (LNs)^5,20^, diminished persistence of systemic micro-metastasis^15^, and a significant increase in overall survival, without concomitantly increasing postoperative events^23^.

The effects of nCRT can be determined by histopathological analysis of surgical resection specimens, assessing the quantity of residual tumor and providing additional information on prognosis and treatment response^22^. Tumor regression grade (TRG) systems aim to categorize the amount of regressive changes^14^. According to Becker et al.^4^, TRG is commonly used for the study of esophageal/GEJ cancer and other gastrointestinal malignancies. In fact, pathological stage assessed after neoadjuvant treatment can give reliable information concerning disease free and overall survival and supports the idea that patients without residual carcinoma in resection specimens have survival advantage when compared to patients with residual carcinoma^6^. In addition to treatment response in primary tumors, the regression *status* of metastatic LNs may be of great prognostic relevance in patients with locally advanced esophageal carcinoma^17^.

In this study, we aimed to investigate the impact on pathological, clinical, and overall survival of neoadjuvant treatment in esophageal/EGJ cancer patients. We also aimed to investigate the possible association between the Becker TRG system and lymph node regression (LNR) and clinicopathological outcomes, including the Clavien-Dindo classification of surgical complications, LN *status*, and the presence of lymphatic and venous invasion. In addition, we analyzed the relationship between the modifications induced by nCRT and overall patient survival.

## METHODS

### Population

All patients diagnosed with SCC or ADC of the esophagus/EGJ (except type III GEJ ADC – Siewert classification), who either received neoadjuvant chemoradiotherapy followed by surgical resection or were exclusively submitted to surgical treatment from January 1, 2006, to December 31, 2020, at the University Hospital Center of Sao Joao (CHUSJ, Porto, Portugal) were selected.

Exclusion criteria included the following: patients under 18 years of age; incomplete information on staging and/or therapy; impossibility of retrieving slides or blocks from surgical specimens; and type III ADC of the GEJ. Our series included 139 patients, of whom 94 belonged to the nCRT group, which is our study's primary interest.

After ethical approval by the CHUSJ/FMUP Ethics Committee for Health (CE 404-21), patient information and clinical data were obtained from hospital databases. Each patient's course of treatment was decided by the assistant physicians, after discussion of each case in multidisciplinary consults.

### Pathological response assessment

Hematoxylin and eosin stained from all surgical specimens were obtained retrospectively from the pathology department of CHUSJ and were reviewed by a pathologist with experience in gastrointestinal pathology (I.G.). TRG of the primary tumor was assessed according to the Becker system^4^, which is based on the assessment of residual tumor cell percentage, as compared to macroscopically identifiable tumor bed. Becker system included three grades:

1. grade 1, complete (0% residual tumor; grade 1a) or subtotal tumor regression (<10% residual tumor; grade 1b);
2. grade 2, partial tumor regression (10–50% residual tumor); and
3. grade 3, minimal or no tumor regression (>50% residual tumor). For statistical purposes, TRGs were divided into two groups: good responders (TRG 1a and TRG 1b) and poor responders (TRG 2 and TRG 3).

**Figure 1:**
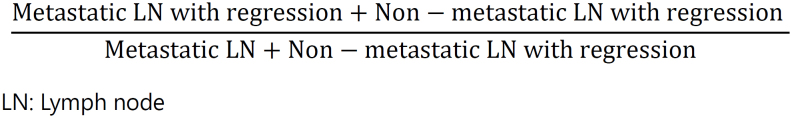
Lymph node regression rate.

Lymph nodes were also evaluated. We assessed the total number of LNs that were retrieved during the surgical procedure, the number of metastatic LNs, as well as regression changes induced by nCRT, both in metastatic and non-metastatic LNs. From these data, the rate of LNR changes was calculated using the formula presented in Figure 1.

### Statistical Analysis

Data were analyzed using IBM SPSS^®^ Statistics version 27. Continuous variables were assessed for normality by visual analysis of their histograms and described using their median and interquartile range. Categorical variables were described with absolute and relative frequencies. Survival times were compared between groups using Kaplan-Meier curves and the Mantel-Cox log-rank test. When modeling survival using continuous variables, the Cox regression analysis was used. Dichotomous outcomes were modeled with binary logistic regression. Simple associations between categorical variables were assessed using Pearson's chi-square test. Differences in the distribution of continuous variables between groups were assessed using the Mann-Whitney U test.

## RESULTS

We studied 139 patients with a median age of 62 years (range: 55–70). The majority of patients were men (n=120; 86.3%). The baseline characteristics of the patients are described in Table 1.

**Table 1:** Sample descriptive statistics.

		Total (n=139)	nCRT (n=96)	No nCRT (n=43)
Age (years), median (range)		62 (55–70)	61.5(55–69)	63(57–72)
Gender, n(%)	Male	120 (86.3)	84 (87.5)	36 (83.7)
	Female	19 (13.7)	12 (12.5)	7 (16.3)
Tumor location, n(%)	Proximal third	5 (3.6)	4 (4.2)	1 (2.3)
	Middle third	57 (41.0)	44 (45.8)	13 (30.2)
	Distal third	66 (47.5)	37 (38.5)	29 (67.5)
	GEJ	11 (7.9)	11 (11.5)	0 (0)
Histological type, n(%)	Adenocarcinoma	33 (23.7)	23 (24.0)	10 (23.3)
	Squamous cell carcinoma	105(75.5)	72 (75.0)	33 (76.7)

GEJ: gastroesophageal junction; nCRT: neoadjuvant chemoradiotherapy.

Of the 139 included patients, 96 (69%) underwent neoadjuvant treatment. Of these, two patients were excluded from TRG analysis because surgical specimen blocks could not be retrieved.

### Pathological outcomes

Regarding anatomopathological outcomes, and more specifically the Becker TRG^4^, among patients who underwent nCRT, 20 (21.5%) showed complete response (TRG 1a), 37 (39.8%) had TRG 1b, 26 (27.9%) had TRG 2, and 10 (10.8%) patients had TRG 3.

When comparing patients who were submitted to nCRT versus patients who did not receive neoadjuvant treatment, we observed an association between nCRT and lymphatic permeation (p=0.034): patients who underwent nCRT had lower rates of lymphatic permeation, but no association was found regarding venous invasion (p=0.18). Furthermore, we verified that, in the group of patients submitted to nCRT (n=94), Becker TRG was associated with both lower lymphatic permeation (p<0.001) and lower venous invasion (p<0.001). These results are summarized in Table 2.

**Table 2:** Association between nCRT and Becker TRG and lymphatic permeation and venous invasion.

		Lymphatic permeation				Venous invasion		
		No (n=92)	Yes (n=47)			No (n=101)	Yes (n=38)	
nCRT	Yes (%)	69 (75)	27 (57.4)	p=0.034	Yes	73 (72.3)	23 (60.5)	p=0.18
	No (%)	23 (25)	20 (42.6)		No	28 (27.7)	15 (39.5)	
		**Lymphatic permeation**				**Venous invasion**		
		**No (n=67)**	**Yes (n=27)**			**No (n=71)**	**Yes (n=23)**	
Becker's grade	TRG 1a (%)	20 (29.9)	1 (3.7)	p=0.001	TRG 1a (%)	20 (28.2)	1 (4.3)	p<0.001
	TRG 1b (%)	29 (43.3)	8 (29.6)		TRG 1b (%)	32 (45.1)	5 (21.7)	
	TRG 2 (%)	14 (20.9)	12 (44.4)		TRG 2 (%)	15 (21.1)	11 (47.8)	
	TRG 3 (%)	4 (6.0)	6 (22.2)		TRG 3 (%)	4 (5.6)	6 (26.1)	

nCRT: neoadjuvant chemoradiotherapy; TRG: tumor regression grade.

Regarding LN invasion, 52 (37.4%) patients presented with metastasis. A Mann-Whitney U test showed that there was a significant difference (U=1163.5; p=0.044) between the number of metastatic LNs and Becker TRG, when patients were divided into good responders (TRG 1a and TRG 1b) and poor responders (TRG 2 and TRG 3). The median number of metastatic LNs for the good responder group was 0 (interquartile range =1) and 1 for the poor responders (interquartile range=2).

The analysis of a possible association between Becker TRG and LNR rate showed no statistical significance (p=0.10).

### Surgical morbidity

Of all patients who underwent surgical treatment, a total of 87 (62.6%) patients presented complications in the postoperative period: 27 (31.0%) had pulmonary complications, 30 (34.5%) gastrointestinal, 14 (16.1%) infectious, 4 (4.6%) cardiovascular, 3 (3.5%) urologic, 1 (1.2%) thromboembolic, and 7 (8.1%) other complications. Notably, 46 (33.1%) patients had complications ≥ grade IIIa, according to the Clavien-Dindo classification of surgical complications^14^. These results are summarized in Table 3.

**Table 3:** Incidence of surgical complications divided into complication groups and according to the Clavien-Dindo classification of surgical complications^14^.

Incidence of surgical complications	
Complication groups	n (87) (%)
Pulmonary	27 (31.0)
Gastrointestinal (GI)	30 (34.5)
Infectious	14 (16.1)
Cardiovascular (CV)	4 (4.6)
Urologic	3 (3.5)
Thromboembolic (TE)	1 (1.2)
Other	7 (8.1)
**Clavien-Dindo classification of surgical complications**	n (87) (%)
Grade I	2 (2.3)
Grade II	39 (44.8)
Grade IIIa	15 (17.2)
Grade IIIb	3 (3.4)
Grade IV	12 (13.8)
Grade V	16 (18.5)

No association was found between the occurrence of surgical complications of any kind and clinicopathological variables when comparing patients treated with nCRT with those not submitted to neoadjuvant treatment (p=0.68). The same was observed when we studied if each individual group of complications had any association with the neoadjuvant treatment.

When studying the nCRT group, among the 58 patients who presented with complications, no association was found between TRG stages and the occurrence of operative complications (p=0.257), as well as between TRG stages and Clavien-Dindo complications ≥ grade IIIa (p=0.869). An analysis was also made in order to look for any association between tumor regression and each complication group. These results are summarized in Table 4.

**Table 4:** Association between Becker TRG (1a, 1b, 2, and 3) and the occurrence of surgical complications in the nCRT group.

	All causes (%)	Clavien-Dindo ≥ grade IIIa (%)	Pulmonary (%)	GI (%)	Infectious (%)	CV (%)	Urologic (%)	TE (%)	Other (%)
TRG 1a	16 (27.6)	6 (20)	4 (21.1)	7 (31.8)	1 (11.1)	2 (66.7)	0 (0)	0 (0)	0 (0)
TRG 1b	23 (39.7)	11 (36.1)	9 (47.4)	7 (31.8)	6 (66.7)	0 (0)	1 (50)	0 (0)	1 (25)
TRG 2	15 (25.9)	10 (33.3)	6 (31.6)	5 (22.7)	1 (11.1)	1 (33.3)	1 (50)	0 (0)	3 (75)
TRG 3	4 (6.9)	3 (10)	0 (0)	3 (13.6)	1 (11.1)	0 (0)	0 (0)	0 (0)	0 (0)
Total	58	30	19	22	9	3	2	0	4
p-value	0.257	0.867	0.381	0.531	0.367	0.180	1.00	N/A	0.287

GI: gastrointestinal; CV: cardiovascular; TE: thromboembolic; TRG: tumor regression grade.

We studied the occurrence of surgical complications as a function of LNR rate. A binary logistic regression showed an odds ratio of 0.784 (p=0.795) for complications of all Clavien-Dindo grades and, interestingly, an odds ratio of 7.19 (p=0.093) for complications ≥ grade IIIa. When analyzing each complication group individually, we found no statistically significant association, although the odds ratio for the occurrence of pulmonary complications was found to be 65.7 (p=0.061).

### Overall survival

**Figure 2:**
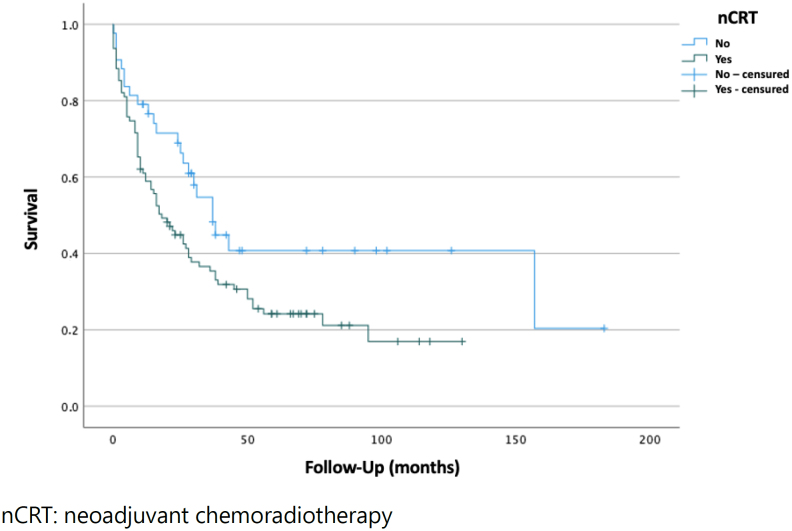
Kaplan-Meier curve, comparing survival time (in months) between patients who received and did not receive neoadjuvant chemoradiotherapy.

Patients who received nCRT had a statistically significant lower median survival when compared to the patients who underwent surgical treatment exclusively (18 [95%CI 10.5–25.45] vs. 37 [95%CI 27.06–46.94] months; p=0.04), portrayed in a Kaplan-Meyer curve (Figure 2).

**Figure 3:**
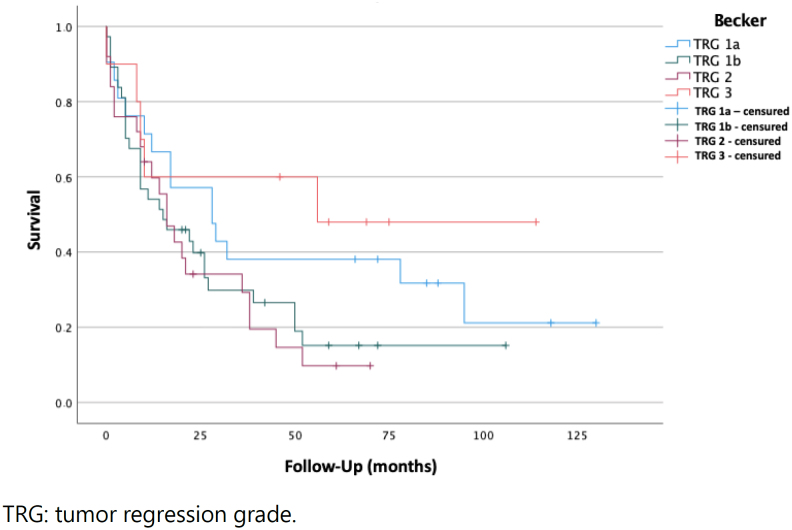
Kaplan-Meier curve, comparing survival time, in months, between Becker tumor regression grade grades 1a, 1b, 2, and 3.

We studied the overall survival of patients who received nCRT according to the TRG grade (TRG 1a: 28 [95%CI 10.06–45.94]; TRG 1b: 15 [95%CI 0–30.01]; TRG 2: 16 [95%CI 9.79–22.21]; TRG 3: 56 [95%CI 32.60–95.68]). Survival analysis did not show any statistical association (Mantel-Cox log-rank, p=0.11). The Kaplan-Meier curve is shown in Figure 3.

**Figure 4:**
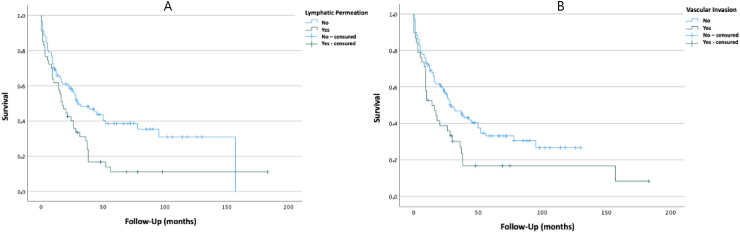
Kaplan-Meier curves, comparing overall survival of patients that present with lymphatic permeation and vascular invasion (A: lymphatic permeation; B: vascular permeation).

Patients who presented with lymphatic permeation had a statistically significant lower median survival (17 [95%CI 10.28–23.71] vs. 30 [95%CI 13.77–46.23] months; p=0.006). Similarly, the patients who presented with venous invasion had a statistically significant lower median survival (14 [95%CI 4.60–23.40] vs. 29 [95%CI 17.83–40.17] months; p=0.024). The Kaplan-Meier curves are shown in Figure 4 (A and B).

Among the patients who presented LN metastases, there was no significant difference in survival between the group that received nCRT and the group that did not, with similar curves for 2 years (overall survival=30%, p=0.282), with a steeper decrease in the survival curve in the group that did not receive nCRT, after the initial 2 years. When comparing overall survival according to the rate of LNs with regression changes, we found the hazards ratio=2.021 (p=0.165), with a tendency to early death when the number of LN with regression was higher.

## DISCUSSION

The role of neoadjuvant chemoradiation in the treatment of patients with locally advanced esophageal or GEJ cancer is nowadays well established, once it has shown to increase tumor downstaging, increased rates of R0 resection, early treatment of subclinical disease and survival benefits, not at the cost of increased rates of unacceptable adverse events^2,8,10,18,23^.

In fact, in the present study, we found no statistical association between being treated with nCRT and the occurrence of any group of postoperative complications, aligning with the findings of Hamai et al.^8^ that nCRT did not cause an increase in morbidity. A more in-depth analysis, focusing on the patients who underwent nCRT treatment, suggested that a higher degree of tumoral regression was not achieved by increasing morbidity, since neither the Becker TRG system nor the LNR rate were associated with postoperative complications. However, when studying how the LNR rate affected postoperative morbidity, we concluded that a higher proportion of regression in the metastatic LNs tends to be related to a higher incidence of Clavien-Dindo complications ≥ grade IIIa and to the occurrence of pulmonary adverse events. Although these findings are not within statistical levels of significance, it might only be because of our study's small sample size. To clarify this point, more studies could be performed with a larger number of patients.

In our series, we verified that there is a statistically significant association between Becker TRG and vascular (lymphatic and venous) invasion in the nCRT group, which several studies have highlighted as having a similar prognostic impact to LN involvement^11^. In fact, we verified that having a lower TRG grade and, therefore, a lower percentage of residual tumor is correlated with lower lymphatic permeation and venous invasion, which is known to provide an advantage in survival^7^.

The analysis of the number of invaded LNs according to the amount of response to the nCRT suggested that a good response to treatment is associated with a lower number of metastatic LNs, which aligns with other findings that LN *status* after neoadjuvant treatment is a key determinant in patient prognosis, even though a pathologically complete response is not curative and the recurrence rate remains significant.^12^

Data from the literature, when comparing groups of patients with equal tumor stages, have underlined that treatment with nCRT followed by surgery provides better overall survival than surgery alone^23^. In our study, patients who were submitted to nCRT showed a statistically significant lower survival. Given the retrospective nature of this research, this can be explained by the fact that in our study, the two groups of patients analyzed did not present with similar initial tumor stages, as the patients who did not receive nCRT were the patients who were initially diagnosed with limited disease (cT1-T2 cN0 M0), which naturally leads to a better baseline prognosis and, therefore, a better overall survival.

Analyzing the overall survival according to TRG stages, the differences found were not statistically significant, although, tendentially, higher percentages of regression lead to a better prognosis. Unexpectedly, in our series, patients with TRG 3 showed a higher overall survival; however, this finding is in all probability due to the very low number of patients in this TRG category.

The overall survival observed in patients according to their lymphatic and venous invasion *status* was as expected, with lower lymphatic permeation and venous invasion being significantly associated with higher rates of overall survival. These findings are in accordance to the results in other recent studies^24^, and therefore, these pathological markers could be further studied in order to solidify their predictive and prognostic value.

There are some limitations to our study. This study is retrospective, and it was conducted at a single hospital center, which can lead to a potential selection bias. Moreover, the sample size is relatively small, which can lead to inaccurate results. In future, considering a multicenter study and, consequently, widening the sample size could result in more accurate statistical findings.

## CONCLUSION

Neoadjuvant chemoradiotherapy is a safe choice of treatment, since it is not associated with higher incidences of postoperative complications. Similarly, different degrees of response to treatment do not seem to affect the rate of complications, as was verified for the rate of LNR. Furthermore, it was significantly associated with lower rates of vascular invasion, which has been associated with a survival advantage, as observed in our series. Additional studies focusing on these pathological findings could solidify their role as valuable indicators of response to treatment, as well as prognostic markers.
